# Evaluation of representativeness and sustainability of PCOS rat models induced by letrozole with or without high-fat diet

**DOI:** 10.1038/s41598-025-15026-4

**Published:** 2025-08-21

**Authors:** Shanqin Qi, Haiyan Yu, Wen Hu, Dongshuai Xia, Qinghan Shi, Kehua Wang

**Affiliations:** 1 The First Clinical Medical College, Shandong University of Traditional Chinese Medicine, Jinan, 250355 Shandong China; 2 Department of Reproductive Medicine, the Affiliated Taian City Central Hospital of Qingdao University, Taian, 271000 Shandong China; 3 Central Laboratory, the Affiliated Taian City Central Hospital of Qingdao University, Taian, 271000 Shandong China; 4 Department of Gynecology, the Affiliated Taian City Central Hospital of Qingdao University, Taian, 271000 Shandong China; 5 Reproduction and Genetics Center, Affiliated Hospital of Shandong University of Traditional Chinese Medicine, Jinan, 250014 Shandong China

**Keywords:** PCOS, Obesity, Rat model, Model recovery, Metabolic disorder, Diseases, Endocrinology

## Abstract

This article aimed to evaluate the representativeness and sustainability of a rat model of polycystic ovary syndrome (PCOS) induced by letrozole (LE) with or without a high-fat diet (HFD). Sexually mature SD rats were randomly divided into a sham group (receiving 1% carboxymethyl cellulose sodium + standard chow, n = 9), a letrozole group (receiving LE + standard chow, n = 15), and a letrozole combined with HFD group (receiving LE + HFD, n = 15). After 21 days, model tests were performed based on body weight, estrous cycle, hormone levels, and ovarian histological changes, and successful modeling rats in LE and LE + HFD groups were further divided into two subgroups: an induction continuation group and an induction termination group (n = 6 in each group), respectively, which were treated for an additional 5 weeks. Changes in body weight, hormone levels, metabolic parameters, vaginal cytology, and ovarian histology were compared among the groups. Following 21 days of induction, the LE group exhibited significant differences in body weight, serum testosterone concentration, estrous cycle, and ovarian tissue morphology. The LE + HFD group showed significant increases in serum lipid and insulin levels. Upon subdivision, the PCOS phenotype in the letrozole continuation induction group (LE-con group) persisted, while it gradually subsided in the termination group (LE-ter group). Body weight, fasting insulin levels, and the homeostasis model assessment of insulin resistance index in the LE + HFD induction continuation group (LE + HFD-con group) were notably higher than those in the LE-con group, and ovarian histology were more severely disrupted. In conclusion, the LE + HFD induced rats more closely mimic the pathological characteristics of clinical PCOS and thus represent a more representative model compared to those induced by LE alone. However, both models tend to recover after discontinuation, indicating that medication should be continued during subsequent treatment to ensure the sustainability of the models.

## Introduction

Polycystic ovary syndrome (PCOS) is a common endocrine disorder, affecting up to 6–20% of women of childbearing age^1^. Characterized by oligo- or anovulation, elevated androgen levels, and polycystic ovarian morphology^2–4^, this syndrome is the leading cause of ovulatory infertility^1,4^. It is often associated with metabolic abnormalities, such as obesity and diabetes mellitus^5,6^, and is closely related to the central nervous system, liver and kidney, cardiovascular and other organs^2,5,7,8^. The specific pathogenesis of PCOS is unclear, and there is currently no ideal treatment in clinical practice.

Research on PCOS has been extensive, including in vivo and in vitro studies. Animal experiments are important means of exploring pathogenesis and evaluating treatment efficacy, and are of great value for revealing the pathophysiological mechanisms of PCOS^9,10^. As a nonsteroidal competitive aromatase inhibitor, letrozole (LE) inhibits the conversion of androgens to estrogens in peripheral tissues^11–13^, thereby increasing androgen levels. LE-induced PCOS rats generally exhibit elevated testosterone (T) and luteinizing hormone (LH), decreased estradiol (E2), increased body weight, disturbed estrous cycles, increased cystic and atretic follicles, and decreased corpus luteum^14–18^, which aligns with the characteristics of patients with PCOS.

Approximately half of PCOS patients are obese or overweight^19^, and obesity, in turn, increases the risk of PCOS by nearly 2–3 times^20,21^. Given that LE-induced rodents have been described as inconsistently exhibiting metabolic abnormalities of PCOS^9,12,22,23^, and since a high-fat diet (HFD) is the common method for inducing obesity and dyslipidemia^10,24,25^, the combination of LE with HFD has become the optimal choice for creating obese PCOS rat models^22,24–27^.

Interventions in animal studies are often necessary to mimic clinical treatments for PCOS, necessitating a stable animal model to ensure efficacy is not compromised. Therefore, we conducted this experiment to assess the sustainability of the model by observing the production and regression of the PCOS phenotype. We compared rats treated with LE combined with HFD to those induced with letrozole alone to explore an animal model that better reproduces clinical PCOS characteristics.

## Methods

### Material

#### Experimental animal

According to the 3R principle of animal experiments, a total of 39 Sprague Dawley rats (6-week old, female), purchased from Zhejiang Charles River Experimental Animal Technology Co., Ltd. (license number: SCXK (Zhejiang) 20240001), were included. They were housed in a specific pathogen-free laboratory at room temperature (20 ± 2) ℃ and relative humidity (55 ± 5)%, with a cycle of 12 h of light/darkness exposure and free access to food and water. This experiment has been approved by the Animal Experiment Ethics Committee of the Affiliated Taian City Central Hospital of Qingdao University (Project number: 2024-06-66). All methods are reported in accordance with ARRIVE guidelines and all procedures were carried out in accordance with relevant guidelines and regulations.

Clinical trial number: Not applicable.

#### Main reagent

Letrozole tablets (Zhejiang Haizheng Pharmaceutical Co., Ltd.); Carboxymethyl cellulose sodium (CMC, Beijing Solarbio Science & Technology Co., Ltd.); Standard chow (Beijing Keaoxieli Feed Co., Ltd., the main nutrients are water, crude protein, crude fat, crude fiber, crude recovery, calcium and phosphorus, etc.); 45% HFD (Ruidi Biotechnology (Shenzhen) Co., Ltd., enriched with lard and soybean oil, energy supply ratio: protein 20%, carbohydrates 35%, fat 45%).

Haematoxylin and eosin (HE) staining solution (Beijing Solarbio Science & Technology Co., Ltd.); Blood glucose monitor (Roche Diagnostic Products (Shanghai) Co., Ltd.); Testosterone, luteinizing hormone, fasting insulin (FINS), triglyceride (TG) and total cholesterol (TC) assay kit (Elabscience Biotechnology Co., Ltd).

## Method

### Animal grouping and experimental protocol

After 1 week of adaptive feeding, the rats with regular estrous cycle were randomly divided into a sham group (n = 9), an LE group (n = 15), and an LE + HFD group (n = 15) after detecting vaginal exfoliated cell smears. Sham group was given 1% CMC solution by gavage daily, 1 ml/100 g/d, and fed with standard chow; LE group was given daily oral administration of letrozole solution (1 mg/Kg/d) with standard rodent chow; LE + HFD group was given daily gavage with letrozole solution and 45% high-fat diet.

After 21 days, 3 rats of were randomly selected for dissection in each of the three groups to evaluate the model quality. The successfully modeled LE group and LE + HFD group rats were then randomly divided into two subgroups respectively: the induction continuation group (LE-con group and LE + HFD-con group, continuing the original modeling protocol) and the induction termination group (LE-ter group and LE + HFD-ter group, both discontinued the original protocol and only fed with normal chow), with 6 animals in each group, and continued the experiment for 5 weeks.

### Body weight

The rats’ weight of each group were monitored every Monday morning at 8:00 am, to compare the differences between the three groups, and adjust the corresponding dose of agents.

### Estrous cycle

From the 11^th^ day of modeling, rats’ vaginal epithelial cells were observed daily. Briefly, to immobilize the rat with the left hand to expose the vaginal opening. With the right hand, gently insert a saline-soaked cotton swab into the vaginal opening and rotate it, apply it evenly to the glass slide, and observe it under the microscope after natural air drying.

Determination of the estrous cycle: Leukocytes are predominant during diestrus stage, nucleated epithelial cells are predominant during proestrus stage, cornered epithelial cells are predominant during estrus stage, and all three types of cells are visible during metestrus stage^28^.

### Specimen collection

After fasting overnight, the caudal vein blood was collected at 8:00 am the next day. Fasting plasma glucose (FPG) was detected by blood glucose meter. Then 2% pentobarbital sodium (40 mg/Kg) was injected intraperitoneally to anesthetize the rats. 5–6 ml of blood was taken from the abdominal aorta and centrifuged at 3000 rpm/min for 10 min after 1 h at room temperature, and the upper serum was collected for testing. Open pneumothorax was used to euthanize the rats before removing bilateral ovaries. When the surrounding adipose tissue was peeled off, the ovaries were fixed in 4% paraformaldehyde solution. Paraffin embedding was carried out 48 h later.

### Determination of sex hormones and metabolic indicators

According to the instructions of ELISA kit (Elabscience Biotechnology Co., Ltd), the concentrations of serum T, LH, FINS, TG and TC were determined respectively, and the homeostasis model assessment of insulin resistance (HOMA-IR) index was calculated according to the formula FPG (mmo/L) * FINS (mIU/L)/22.5.

### Ovarian histology

Wax blocks of ovarian tissue were serially sectioned at a thickness of 5 um. After HE staining, images of ovarian sections were taken using OLYMPUS cellSens Standard software, and the number of growing follicles at each stage, cystic follicles, atresia follicles, and corpus luteum was counted under a 4 × microscope.

### Statistical methods

SPSS 27 software was used for statistical analysis. The normality test and the homogeneity test of variance were performed first, and numerical variables conforming to the normal distribution were expressed by mean ± standard deviation ($\overline{\text{x} }$ ± s). Significance test was performed by one-way ANOVA, with LSD test for post hoc analysis, among groups. Significance of non-normally distributed numerical variables was tested by Mann–Whitney U test. Graphpad prism 9.5 software was chosen for the diagrams. *P* < 0.05 was considered statistically significant.

## Results

### Model evaluation

#### Body weight comparison

**Fig. 1:**
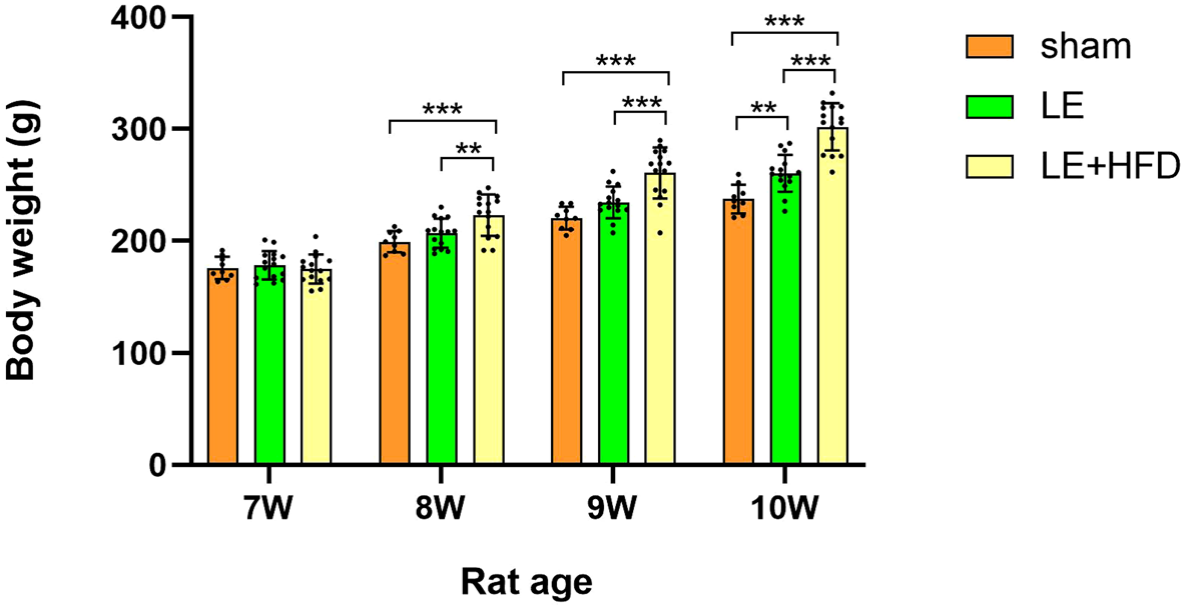
Comparison of body weights during model building. *P < 0.05, **P < 0.01, ***P < 0.001. LE, letrozole; HFD, high-fat diet.

There was no significant difference in body weight between the rats at the end of adaptive feeding (7-week old, *P* > 0.05). After 1 week of modeling (8-week old), the weight of LE + HFD group increased rapidly, which was significantly higher than that of sham group and LE group (222.99 ± 18.48 vs. 199.21 ± 9.53, *P* < 0.001; 222.99 ± 18.48 vs. 206.79 ± 12.95, *P* = 0.005), and the difference was more pronounced in the next two weeks (both *P* < 0.05). The weight gain of LE group was smaller when compared with LE + HFD group, and was significantly higher than that of sham group at the end of modeling (260.39 ± 16.54 vs. 237.39 ± 12.73, *P* = 0.004). See Fig. 1.

#### Estrous cycle evaluation

**Fig. 2:**
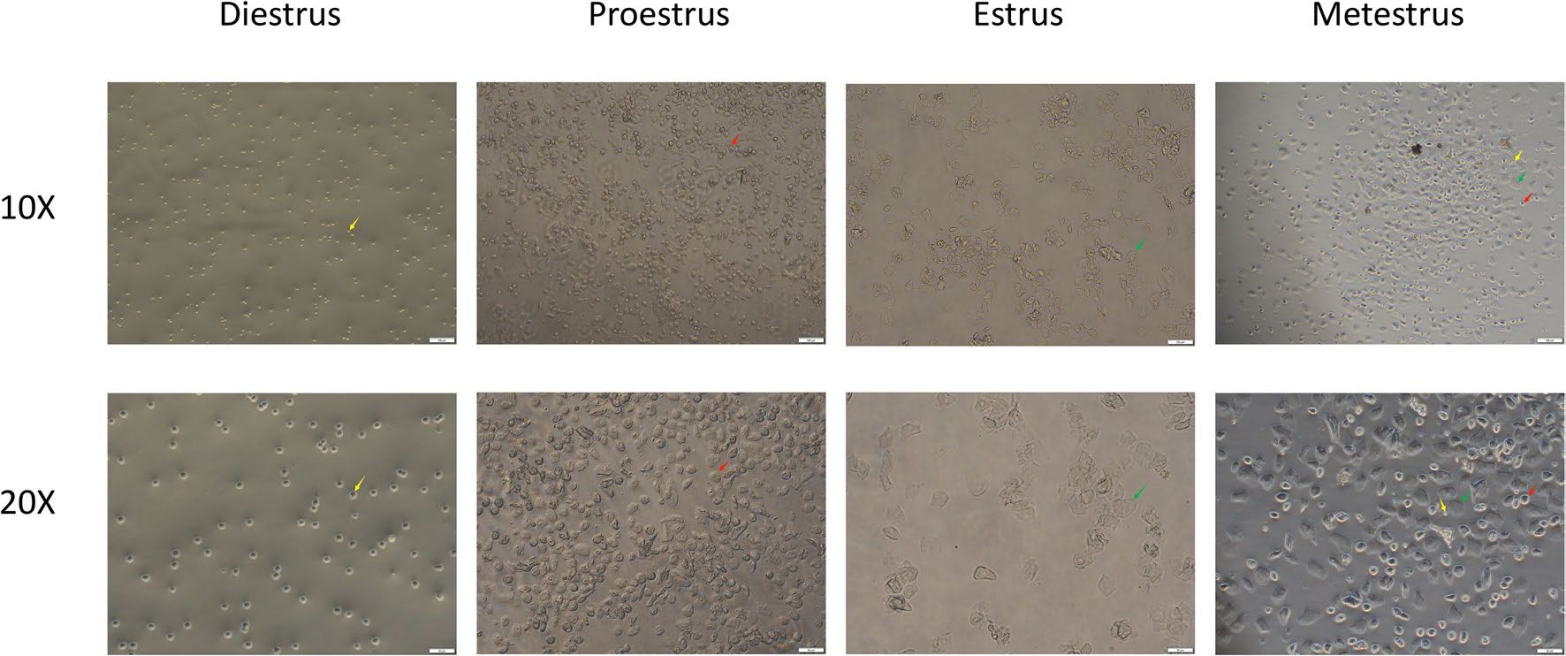
Rat vaginal cell smear. Magnify 10x and 20x, respectively. L, leukocytes (yellow arrows); N, nucleated dpithelial cells (red arrows); C, cornified squamousepithelial cells (green arrows).

Continuous vaginal cytology on the 11th to 21 st days of modeling showed that the rats in the sham group maintained a regular estrous cycle every 4–5 days, while the LE group and LE + HFD group lost their regularity, showing prolonged or disordered estrous cycle and decreased proestrus and estrus (Fig. 2).

#### Sex hormone measurement

**Fig. 3:**
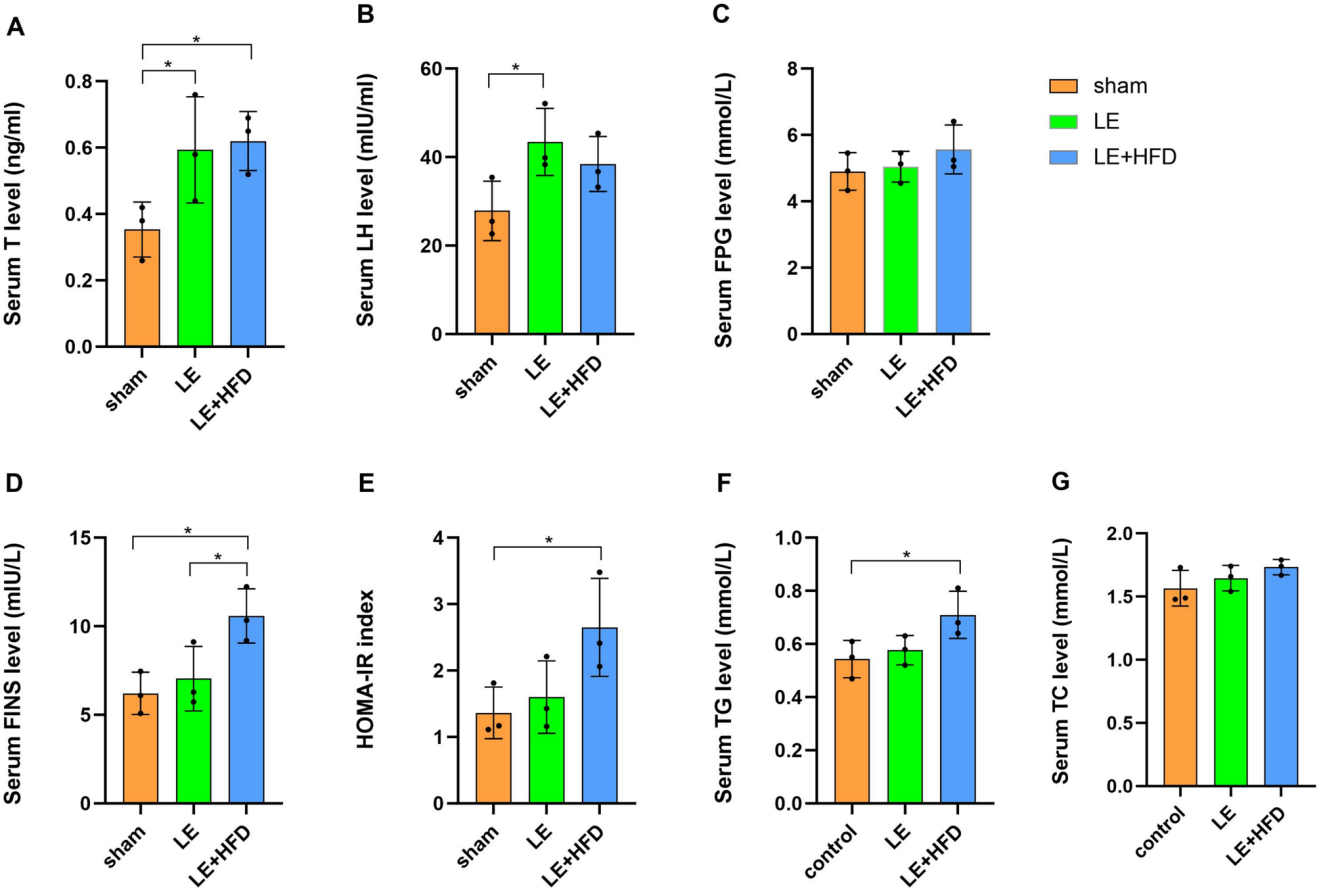
Serological results of rats at the end of modeling. (**A**-**B**) Comparison of T/LH levels. (**C**-**E**) Comparison of FPG/FINS/HOMA-IR levels. (**F**-**G**) Comparison of TG/TC levels. *P < 0.05, **P < 0.01, ***P < 0.001. T, testosterone; LH, luteinizing hormone; FPG, fasting plasma glucose; FINS, fasting insulin; HOMA-IR, homeostasis model assessment of insulin resistance; TG, triglyceride; TC, total cholesterol.

After letrozole treatment, T concentrations in LE group and LE + HFD group were significantly higher than those in sham group (0.59 ± 0.16 vs. 0.35 ± 0.08, *P* = 0.045; 0.62 ± 0.09 vs. 0.35 ± 0.08, *P* = 0.031), but were similar between LE group and LE + HFD group (*P* = 0.788) (Fig. 3A). LH was elevated in both model groups, but only the difference between LE group and sham group was significant (43.44 ± 7.56 vs. 27.85 ± 6.71, *P* = 0.032) (Fig. 3B).

#### Glycolipid metabolism examination

After 21-d induction, FPG levels in the three groups were parallel (4.90 ± 0.57 vs. 5.05 ± 0.46 vs. 5.57 ± 0.74, *P* = 0.415) (Fig. 3C). FINS of LE group and sham group were comparable (7.04 ± 1.82 vs. 6.21 ± 1.19, *P* = 0.531) (Fig. 3D), which were both significantly lower than that of LE + HFD group (7.04 ± 1.82 vs. 10.59 ± 1.53, *P* = 0.030; 6.21 ± 1.19 vs. 10.59 ± 1.53, *P* = 0.013). Correspondingly, HOMA-IR of LE + HFD group was the highest, which was significantly different from that of sham group (2.65 ± 0.74 vs. 1.36 ± 0.39, *P* = 0.034), but not statistically significant when compared with LE group (2.65 ± 0.74 vs. 1.60 ± 0.55, *P* = 0.067) (Fig. 3E), suggesting that only hypersecretion of insulin could control blood glucose in LE + HFD group.

TG level of LE + HFD group was significantly higher than that of sham group (0.71 ± 0.09 vs. 0.54 ± 0.07, *P* = 0.031), but not significant with LE group (0.71 ± 0.09 vs. 0.58 ± 0.06, *P* = 0.066), and the latter was equivalent to sham group (*P* = 0.595) (Fig. 3F). TC level of the two model groups was slightly higher than that of the sham group, and there was no statistical difference among the three groups (1.57 ± 0.14 vs. 1.65 ± 0.10 vs. 1.73 ± 0.06, all *P* > 0.05) (Fig. 3G).

#### Ovarian HE staining assessment

**Fig. 4:**
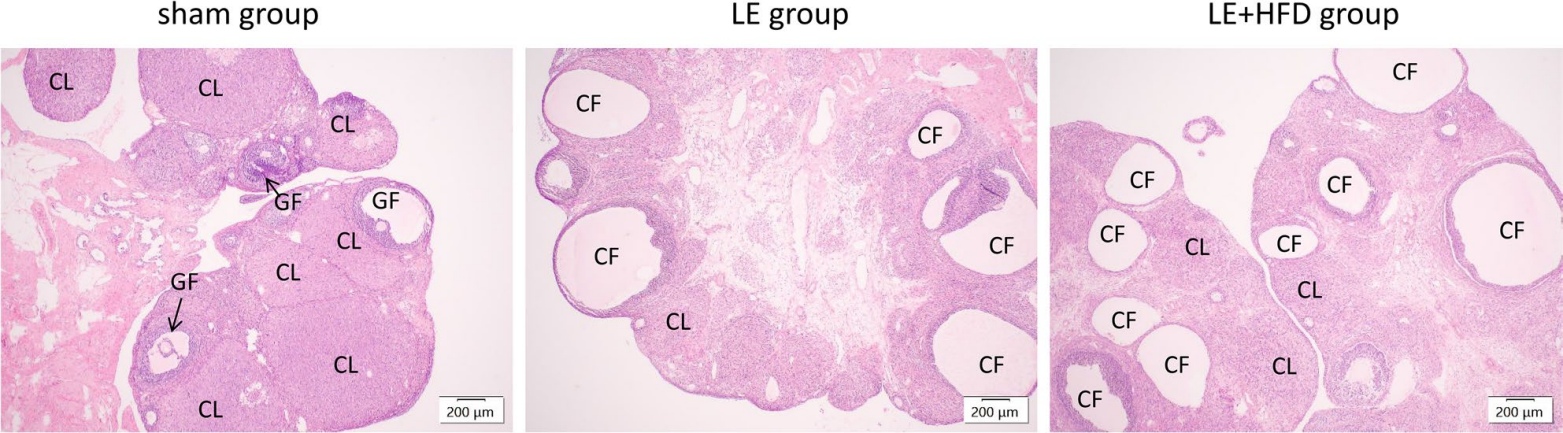
HE-stained ovarian tissue sections of rats at the end of modeling. Magnify 4x. CF, cystic follicles; CL, corpus luteum; GF, growth follicles.

HE stained sections of ovarian tissue had follicles of various developmental stages in the sham group, surrounded by multilayer granulosa cells (GCs), and mature follicles and corpus luteum representing ovulation were visible. In the two model groups, ovarian cortex was thickened, and follicles were cystically dilated, surrounded by fewer sparsely arranged GCs. Corpus luteum decreased in number and size, and follicular atresia degeneration was common (Fig. 4).

### Parameters after modeling

#### Changes in body weight

**Fig. 5:**
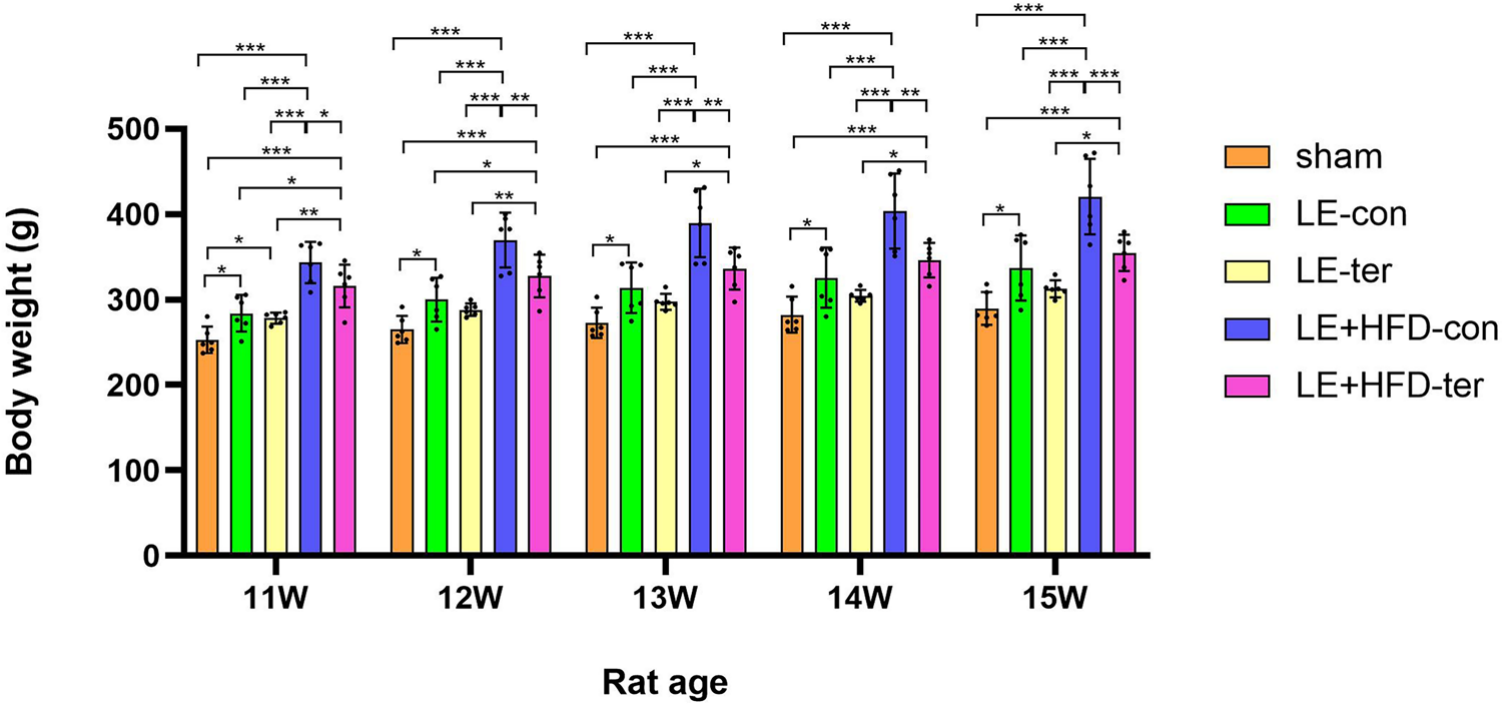
Comparison of body weights at the end of the experiment. *P <0.05, **P < 0.01, ***P < 0.001.

In the following studies, weight gain slowed in the two subgroups that discontinued dosing. The statistical significance between LE-ter group and sham group disappeared at week 12 (288.27 ± 7.34 vs. 265.00 ± 15.91, *P* = 0.091), while LE + HFD-ter group still maintained a significant advantage over LE-con group (327.53 ± 25.02 vs. 300.03 ± 25.73, *P* = 0.048), until week 13 (336.40 ± 24.68 vs. 313.85 ± 29.61, *P* = 0.152). See Fig. 5.

#### Changes of estrous cycle

**Fig. 6:**
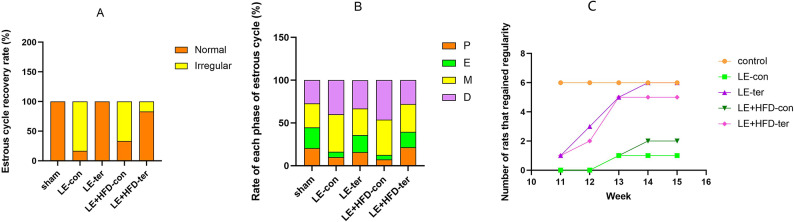
Estrous cycle monitoring. A: Estrous cycle recovery rate. B: Rate of each phase of estrous cycle. C: Number of rats that regained regularity after modeling. P, proestrus; E, estrus; M, metestrus; D, diestrus

Vaginal cytology for 35 consecutive days after modeling showed that one rat in LE-con group resumed regular estrous cycle (4-5-d estrous cycle, 3 consecutive cycles or more) in the third week (13-week old), with a recovery rate of 16.67%. All LE-ter groups regained their regularity, with 1 in the first week, 2 in the second week, 2 in the third week, and 1 in the fourth week. The LE + HFD-con group achieved a recovery rate of 33.33%, with one in the third week and one in the fourth week. 5 rats recovered in LE + HFD-ter group, up to 83.33%, including 1 in the first week, 1 in the second week, and 3 in the third week (Fig. 6A-C).

#### Comparison of hormone levels

**Fig. 7:**
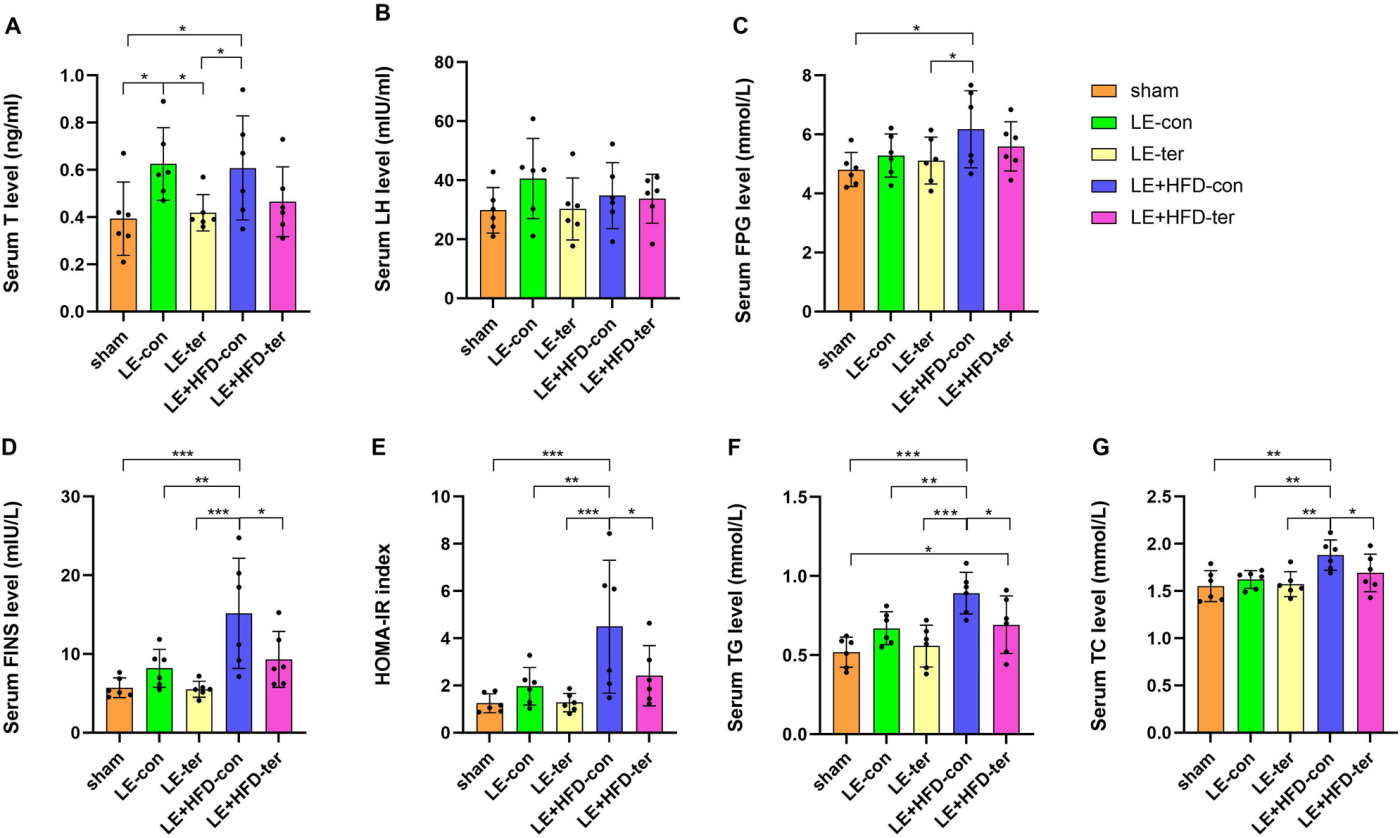
Serological results at the end of the experiment. (**A**–**B**) Comparison of T/LH levels. (**C**–**E**) Comparison of FPG/FINS/HOMA-IR levels. (**F**–**G**) Comparison of TG/TC levels. *P < 0.05, **P < 0.01, ***P < 0.001. T, testosterone; LH, luteinizing hormone; FPG, fasting plasma glucose; FINS, fasting insulin; HOMA-IR, homeostasis model assessment of insulin resistance; TG, triglyceride; TC, total cholesterol.

At the end of the experiment, serum T level in LE-con group was higher than that in LE-ter group and sham group (0.63 ± 0.15 vs. 0.42 ± 0.08, *P* = 0.032; 0.63 ± 0.15 vs. 0.39 ± 0.16, *P* = 0.017), not significant with that in LE + HFD-ter group (0.63 ± 0.15 vs. 0.47 ± 0.15, *P* = 0.090). LE + HFD-con group held similar T to LE-con group (0.61 ± 0.22 vs. 0.63 ± 0.15, *P* = 0.856), but was significantly superior to LE-ter group and sham group respectively (*P* = 0.047; *P* = 0.026) (Fig. 7A).

LH showed similar levels in all groups (29.80 ± 7.70 vs. 40.57 ± 13.61 vs. 30.26 ± 10.51 vs. 34.75 ± 11.22 vs. 33.74 ± 8.30, *P* = 0.413) (Fig. 7B).

#### Comparison of biochemical indicators

FPG of LE + HFD-con group was the highest, which had a significant advantage over sham group and LE-ter group (6.18 ± 1.31 vs. 4.81 ± 0.58, *P* = 0.013; 6.18 ± 1.31 vs. 5.12 ± 0.79, *P* = 0.048) (Fig. 7C). FINS and HOMA-IR extended this trend, LE + HFD-con group significantly outperformed the other 4 parallel groups (all *P* < 0.05), indicating further reduced glucose responsiveness to insulin (Fig. 7D and E).

TG content of LE + HFD-con group far exceeded that of the other four groups (0.89 ± 0.13 vs. 0.52 ± 0.10 vs. 0.67 ± 0.10 vs. 0.56 ± 0.13 vs. 0.69 ± 0.18, all *P* < 0.05), sham group had significantly lower TG than LE + HFD-ter group (0.52 ± 0.10 vs. 0.69 ± 0.18, *P* = 0.033), but not statistically different from those in LE-con group (0.52 ± 0.10 vs. 0.67 ± 0.10, *P* = 0.059) (Fig. 7F).

TC level in LE + HFD-con group was much higher than that in other groups (1.88 ± 0.16 vs. 1.55 ± 0.16 vs. 1.62 ± 0.09 vs. 1.57 ± 0.13 vs. 1.69 ± 0.20, all *P* < 0.05), while the other four groups were comparable (all *P* > 0.05) (Fig. 7G).

#### Ovarian histological changes

**Fig. 8:**
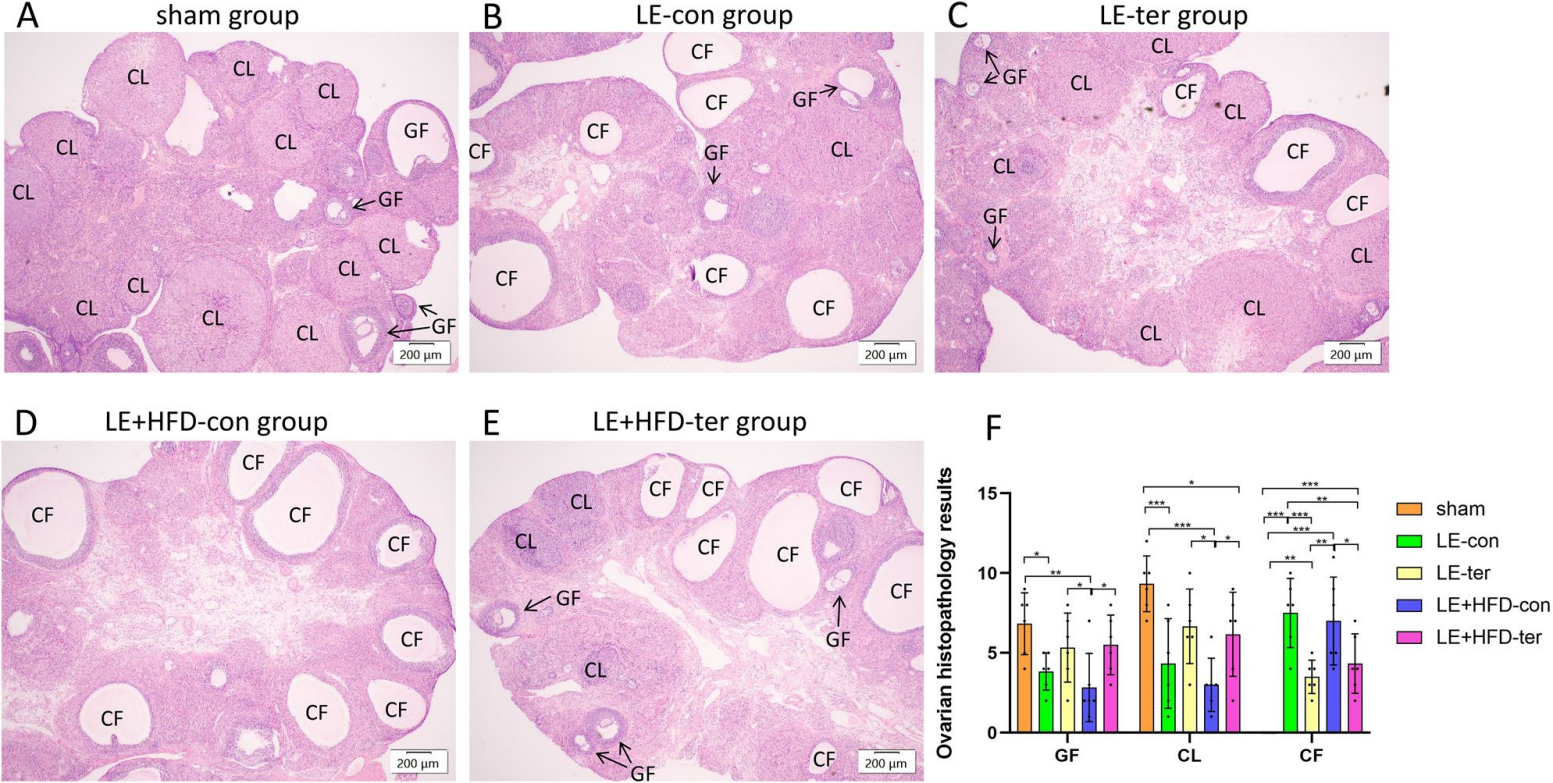
HE-stained ovarian tissue sections of rats at the end of the experiment. A-E: Picture of HE staining of ovarian sections. Magnify 4x. F: Comparison of the number of follicles and corpus luteum. *P < 0.05, **P < 0.01, ***P < 0.001. CF, cystic follicles; CL, corpus luteum; GF, growth follicles; AF, atretic follicles.

As shown in Fig. 8, the number of growing follicles in LE + HFD-con group was much smaller than that in LE + HFD-ter group, LE-ter group and sham group (2.83 ± 2.14 vs. 5.50 ± 1.87 vs. 5.33 ± 2.16 vs. 6.83 ± 1.94, all *P* < 0.05), but comparable to that in LE-con group (2.83 ± 2.14 vs. 3.83 ± 1.17, *P* = 0.368). LE-con group had significantly decreased growing follicles than sham group (3.83 ± 1.17 vs. 6.83 ± 1.94, *P* = 0.011). No significant differences were detected among LE-ter group, LE + HFD-ter group and LE-con group, and consistently, among the first two groups and sham group (all *P* > 0.05).

The corpus luteum number in LE-ter group and LE + HFD-ter group was approximated (6.67 ± 2.34 vs. 6.17 ± 2.64, *P* = 0.708), which was significantly improved compared with their corresponding continuation subgroups. LE-ter group was close to sham group (6.67 ± 2.34 vs. 9.33 ± 1.75, *P* = 0.054), while the number of corpus luteum in LE + HFD-ter group was significantly lower than that in sham group (6.17 ± 2.64 vs. 9.33 ± 1.75, *P* = 0.024).

Cystic follicles were absent in the ovaries of sham group, but were abundant in LE-con group, followed by LE + HFD-con group, both were far more than LE-ter group and LE + HFD-ter group (all *P* < 0.05).

## Discussion

The large number of PCOS patients, many of whom are of childbearing age, are suffering from amenorrhea, infertility, obesity, acne, and other problems that seriously affect reproductive function and quality of life^4,5,29^. In response to this dilemma, experts have conducted a lot of research. Due to ethical and material access restrictions on human trials, researchers turn to animals to find alternatives. Considering factors such as economy, experimental period and effect, rodents are currently the best choice^13,30^. Compared with mice, rats are larger, easier to operate and obtain materials, and have a stable genetic background, which makes them commonly used for reproductive research^13,31^. Meanwhile, PCOS is a disease with a combination of endocrine and metabolic disorders, and clinical patients are often obese^19,27^. Both hyperandrogenism and hyperlipidemia have prominent negative effects on female reproduction^10,27,32^. Therefore, the rat model should take into account both endocrine and metabolic phenotypes. LE combined with HFD has been shown to demonstrate the endocrine and metabolic disorder phenotypes of PCOS well and is a common modeling method for obese PCOS models^22,24,26,27^.

It is well known that the 4–5 day estrous cycle of rats, the structure of a double uterus, the shorter gestation cycle, etc., make them far more fertile than humans^31,33^. Therefore, it is doubtful whether rat models induced by drugs maintain a stable PCOS phenotype. As reported, 3-week-old female rats who were administered low- (1200 ug/Kg/d) or high-dose (1200 ug/Kg/d) fatrozole for 60 days successfully inhibited estrous cycle^34^. However, some rats resumed within 1 week, and all resumed within 30 days after drug withdrawal, regardless of dose, and the pregnancy outcome of mating with normal male rats was no different from that of normal female rats, indicating rapid recovery of fertility. Since many interventions are performed on rat models, model consistency during the period is a prerequisite. To seek for a suitable regimen, we employed LE or LE combined with HFD to induce PCOS rats, which were then divided into dose continuation and termination subgroups to compare the sustainability of the model.

According to our data, 3 weeks of letrozole with or without HFD disrupted the regular estrous cycle of rats, significantly elevated serum T levels, and ovarian sections showed polycystic changes and luteal absence, aligning with the clinical diagnosis of PCOS^35^. Although many studies^14,36–38^ confirmed that letrozole can increase body weight and lipid level in rats, according to our data, the effect of letrozole is far less than that directly driven by HFD, and fat accumulation is difficult to eliminate in a short time. This was confirmed in LE + HFD-ter group, where 3 weeks of letrozole and HFD resulted in significant increases in body weight and lipid levels, which declined slowly after discontinuation and were consistently higher than those in LE-con group with letrozole exposure alone, indicating that the combination of HFD can cause lipid metabolism.

Androgen dysregulation is considered the main culprit of PCOS, with clinical or biochemical hyperandrogenism occurring in 80–90% of patients^30,39^. High doses of androgens recruit excess early follicles, promote anti-Müllerian hormone (AMH) secretion by GCs, and arrest follicular growth^40^, so controlling androgens improves the ovarian microenvironment and promotes follicular development. Our data indicated a gradual resolution of hyperandrogenism after discontinuation, but obesity did not seem to contribute to higher T concentrations. This is inconsistent with many studies^41–43^, which demonstrated that T levels were positively correlated with BMI and fat mass, and that obesity PCOS patients had higher free testosterone and free androgen indices. However, we got support in another study^10^, who established a puberty rat model with DHEA, and did not form a difference in T with or without HFD, and no significant changes in androgens were observed in rats that received HFD alone for 3 or 6 weeks. For this inconsistency, we believe that although obesity exacerbates hyperandrogenism through different mechanisms^2,44^, this indirect effect becomes less prominent in the context of direct drug exposure such as androgen or letrozole.

Pharmacologically, letrozole inhibits androgen conversion to estrogen, and decreased E2 is negatively fed back to the HPO axis, promoting gonadotropin-releasing hormone (GnRH) secretion, primarily LH secretion^11,22^. Most studies^15,18,25,36,45^ induced significantly elevated LH in rats by letrozole, but some scholars^46,47^ detected decreased LH. Obesity has been reported to impair LH secretion, which is due to elevated E2 synthesis in adipose tissue and circulating hyperleptinemia both negative feedback suppression of GnRH/LH pulses, and disruption of LH peaks has been observed in high-fat fed rats^24,48,49^. We believe that, besides the effects of obesity, the characteristic secretion pattern of LH may be a major factor in inconsistent results. LH is pulsatile release, and the amplitude of LH surge before ovulation can reach 4–5 times the basal levels, so a single serum measurement may not accurately reflect LH concentration, and regular monitoring of intravenous catheters in the preestrous period may be more valuable^24^.

Hyperandrogenism often leads to insulin resistance (IR) in patients with PCOS^3^, and testosterone level has been positively correlated with insulin levels in studies^50^. Hyperinsulinemia occurs in the context of IR, when peripheral tissue responsiveness to insulin decreases^51^ and islet β cells need to release more insulin to maintain glycemic stability^52^. We confirmed that obesity facilitated IR, aggravated metabolic disorders, and prolonged the course of the disease. IR has been reported to be one of the core mechanisms of PCOS, affecting 50–70% of PCOS patients and up to 95% of obese women^3,53^. Besides, it leads to type 2 diabetes, dyslipidemia and metabolic syndrome^7,54,55^. Combined with previous hormone and lipid results, it can be concluded that obesity worsens PCOS phenotype through IR, making metabolic and endocrine disorders more complex to crack.

Obese PCOS is not just a simple superposition of PCOS and obesity, but involves metabolic disorders and chronic inflammation that exacerbate hormonal imbalances, hypothalamus-pituitary-ovary (HPO) axis disorders, and ovulation abnormalities^3,44,56,57^, leading to intractable PCOS pathological processes and complications, and more onerous treatment^58^. This tendency was mapped in our obese rat model and also reflected better similarity and representativeness of LE + HFD rats to human PCOS, a model that compensated for metabolic impairments not achieved with letrozole alone.

Estrous cycle monitoring is a simple and effective method to judge the sexual axis function of rats. Estrous cycles of more than 3 consecutive 4–5 days are regarded as regular cycles^5^. Rats in our model group showed prolonged or irregular cycles. In the next 35-day experiment, regardless of whether letrozole was combined with HFD or not, the sexual cycle tended to resume after stopping the drug. Surprisingly, some rats of the two subgroups that continued to induction also regained regularity. We speculate it to be a contribution of the experimental design: Our rats are sexually mature at the start of the experiment, and PCOS phenotype is completely dependent on admixture induction, so once-daily dosing may not guarantee stable blood concentration. As rats age, their drug sensitivity decreases, resulting in poor model maintenance^31^. Conversely, clinical PCOS is more complex and persistent with the onset of adolescence and throughout adulthood^59^. The number of regular cycles between LE-ter group and LE + HFD-ter group was comparable to that of sham group, indicating the restoration of fertility after 5 weeks of drug withdrawal. If treatment is administered during this period, it is difficult to tell whether the efficacy is partially attributable to the recovery of the model itself, which affects the reliability of the results. Therefore, regardless of the scheme, not only continuation of dosing is a requirement for the post-model treatment process, but also regular monitoring to detect and eliminate non-compliant rats in a timely manner.

After recruitment, development, and dominance of antral follicles, luteal formation marks ovulation and is a prerequisite for successful conception^60^. The number of cystic follicles and corpus luteum in LE-ter group returned to close to that of sham group, suggesting better ovulation, which can also be supported by a 100% cycle reconstruction rate. Combined with unoptimistic histological manifestations and the above-mentioned endocrine changes in LE + HFD-con group, it can be inferred that obesity aggravates ovarian microenvironment defects in PCOS, brings more profound reproductive toxicity, is less prone to dominant follicles and spontaneous ovulation, and impairs conception^40,44,56,61^.

In summary, we have discussed the characteristics of letrozole-related PCOS models, which are prevalent in PCOS research. To the best of our knowledge, this is the first study to examine the sustainability of LE and LE + HFD-induced rat models of PCOS, concluding that LE + HFD regimen more accurately mimics the characteristic profile of human PCOS. For animal studies of PCOS in the field of obstetrics and gynecology, the rat model of this protocol is a suitable choice. This is not only due to the simple administration method and ease of handling in rats, but also because of its reliable quality and comprehensive phenotype. Only by performing drug intervention on a stable model can the baseline be unified and variables controlled, the longer model persistence can accommodate the needs of various experimental periods. It should be noted that the establishment and regression of the PCOS phenotype in sexually mature rats is relatively rapid and highly drug-dependent. By comparing subgroups that continued and ceased the administration, we found that rats exhibited uncontrollable differences in body weight, androgen levels, glycolipid metabolism, recovery from the estrous cycle, and changes in ovarian histology. Therefore, continued dosing is essential during the intervention stage after modeling to ensure the consistency of the model.

There are some limitations to this study. Firstly, for the model-building scheme, we employed a clinically analogous intragastric administration method, which was not compared with other methods such as injection or subcutaneous sustained-release granules, potentially limiting the comprehensiveness of our summary of the letrozole-induced PCOS model. Secondly, due to the difficulty in obtaining serum samples from living rats, T and LH were only measured at model establishment and sacrifice, lacking dynamic observation during model maintenance. Finally, rat models with prenatal or adolescent androgen exposure may more closely resemble clinical PCOS, which is an area of interest for further research.

## Data Availability

Data are available from the corresponding author for reasonable reasons.
